# Clinicopathological and prognostic significance of programmed death ligand-1 expression in breast cancer: a meta-analysis

**DOI:** 10.1186/s12885-017-3670-1

**Published:** 2017-10-17

**Authors:** Hye Min Kim, Jinae Lee, Ja Seung Koo

**Affiliations:** 1 Department of Pathology, Yonsei University College of Medicine, Severance Hospital, 50 Yonsei-ro, Seodaemun-gu, Seoul, 120-75 South Korea; 2 Biostatistics Collaboration Unit, Yonsei University College of Medicine, Seoul, South Korea

**Keywords:** PD-L1, Breast cancer, Prognosis, Meta-analysis

## Abstract

**Background:**

Programmed cell death-ligand 1 (PD-L1) may be a useful molecule for targeted immunotherapy. Therefore, this meta-analysis aimed to investigate PD-L1 expression in breast cancer and its associations with clinicopathological factors and outcomes, which may help determine whether PD-L1 expression is a useful prognostic marker.

**Methods:**

The Medline Ovid, Cochrane, PubMed, Google Scholar, and Web of Knowledge databases were searched for studies that evaluated the prognostic or clinicopathological significance of PD-L1 expression in patients with breast cancer, and reported at least one survival-related outcome.

**Results:**

Six studies that included 7877 cases were selected for the analysis. Higher PD-L1 expression in all cells was related to higher histological grade and lymph node metastasis. Higher PD-L1 expression in tumor cell was related to larger tumor size, estrogen receptor negativity, progesterone receptor negativity, human epidermal growth factor type-2 positivity, and triple-negative breast cancer. PD-L1 positivity in all cells was associated with poorer disease-free survival, although it was not significantly associated with overall survival.

**Conclusion:**

The present meta-analysis revealed that cases of breast cancer with PD-L1 positivity in all cells exhibited higher histological grades, lymph node metastasis, and poorer disease-free survival. Therefore, positive expression of PD-L1 may be a useful prognostic marker in breast cancer.

**Electronic supplementary material:**

The online version of this article (10.1186/s12885-017-3670-1) contains supplementary material, which is available to authorized users.

## Background

Breast cancer is the most prevalent cancer among women, and is the second leading cause of cancer-related deaths. Molecular alterations are known to affect cancer occurrence and metastasis, which has led to the development of hormonal therapy that targets the estrogen receptor (ER), progesterone receptor (PR), or human epidermal growth factor type 2 (HER-2). However, up to 20% of patients with breast cancer experience disease progression and death, which highlights the need for more effective therapy [1].

The efficacy of immunotherapy is clear for immunogenic tumors, such as malignant melanoma, non-small cell lung cancer, and urothelial carcinoma. Furthermore, programmed cell death protein-1 (PD-1) and programmed cell death-ligand 1 (PD-L1) may be useful molecules for targeted immunotherapy. PD-1 is a co-inhibitory receptor that belongs to the CD28/CTLA-4 family, and serves as a negative regulator of the immune system by inhibiting the function of T-cells in local tissues [2, 3]. PD-L1 (also known as CD275 and B7-H1) is one of the PD-1 ligands and is expressed in tumor cells. The interaction between PD-L1 and PD-1 affects the antitumor immune response and leads to tumor cell proliferation and metastasis [4, 5]. Although breast cancer has not been traditionally considered an immunogenic tumor, several studies have suggested that patients with breast cancer exhibit a defect in their immune response [6, 7]. Furthermore, cases of triple-negative breast cancer (TNBC) or basal-like breast cancer exhibit prominent infiltration of inflammatory cells, which suggests that an altered immune pathway plays a role in tumorigenesis.

Several previous studies have evaluated the role of PD-L1 as a prognostic marker. For example, Zhang et al. evaluated patient with 12 types of epithelial-originated cancers (e.g., breast cancer, cervical cancer, and renal cell carcinoma), and found that PD-L1 positivity was associated with poorer overall survival (OS), compared to PD-L1 negativity [8]. However, several other studies have reported conflicting results [9, 10]. Moreover, regarding the prognosis and PD-L1 immunohistochemical expression in breast cancer, only a data from a single center is available, but those data also provided inconsistent results [11–16]. Therefore, the present meta-analysis aimed to investigate PD-L1 expression in breast cancer and its associations with clinicopathological factors and outcomes. This information may help determine whether PD-L1 expression is a useful prognostic marker.

## Methods

### Literature search and selection criteria

On April 1, 2016, we searched several international databases (Medline Ovid, Cochrane, PubMed, Google Scholar and Web of Knowledge) using the following terms: ‘breast cancer or breast carcinoma’, ‘PD-L1 or B7-H1’, and ‘prognosis’. Two independent researchers (JSK and HMK) reviewed the search results. The inclusion criteria were: (1) studies that evaluated the prognostic or clinicopathological significance of PD-L1 expression in patients with breast cancer, and reported at least one survival-related outcome (disease-free survival [DFS], OS, or survival rates calculable using the article’s data); (2) studies that used an anti-PD-L1 antibody for the immunohistochemistry; and (3) the specimens were obtained using core needle biopsy or from the postoperative specimen. The exclusion criteria were: (1) studies that included patients who had received neoadjuvant chemotherapy; (2) studies that included <50 cases; and (3) studies that were not published in English. The whole text was reviewed when the report fulfilled the inclusion criteria. In cases of disagreement, the reviewers discussed the report and tried to reach a consensus. A third researcher was consulted to provide a final opinion in cases where a consensus could not be reached.

### Data collection

Data extraction was performed according to the Cochrane guidelines. The following variables were extracted for the present meta-analysis: first author’s name, publication year, patients’ nationality, number of patients, trial design, mean age, clinicopathological parameters, PD-L1 positivity, study end-points (DFS and/or OS), and hazard ratios (HR) and 95% confidence intervals (CI). All included studies indicated that written informed consent had been obtained from the included patients.

### Statistical analysis

Q statistics from the chi-square test were used to evaluate the presence of heterogeneity. However, as Q statistics are not very powerful for evaluating heterogeneity, a higher significance level is used to compensate for the low power of the test [17]. The study effects were tested using a random-effect model if the *p*-value from the Q statistic was <0.1 and a fixed-effect model was used if the p-value was ≥0.1. The I^2^ value was also used to evaluate heterogeneity; I^2^ is defined as 100% × ([Q – df] / Q), and ranges between 0% (minor heterogeneity) to 100% (severe heterogeneity), where df = (the number of studies – 1). The standard cut-off values for I^2^ are 25% (low), 50% (moderate), and 75% (high) [18, 19]. For our analyses, we reported relative risks (RRs) with 95% CIs for the clinicopathological factors, and HRs with 95% CIs for DFS and OS. Publication bias was assessed using a funnel plot and Egger’s test. Begg’s test was not considered for the analysis, as it has a very low power for detecting bias in a small sample of studies [20]. All analyses were performed using Comprehensive Meta-Analysis software (version 2.0; Biostat Inc., Englewood, NJ) and R software (version 3.2.2; http://www.r-project.org).

## Results

### Characteristics of the included studies

**Fig. 1:**
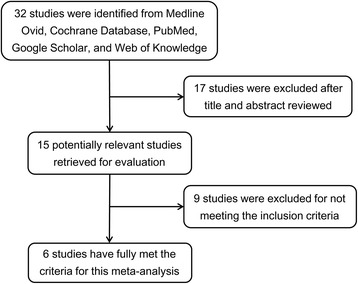
Flow chart of the literature search and study selection

Thirty-two studies were identified from literature search and 17 studies were excluded after title and abstract reviewed. Nine studies were excluded for not meeting the inclusion criteria. Finally, this meta-analysis included 6 studies and 7877 cases [11–16] (Fig. 1). The primary characteristics of the included studies are presented in Additional file 1. Table 1 and Table 2 show the basic characteristics and clinicopathologic parameters of the included studies. The reports were published between 2007 and 2016, and included patients from China, Brazil, England, Switzerland, Korea, and Saudi Arabia. In 3 of the 6 studies, molecular genetic subtypes were analyzed. However, among the 4578 cases included, 2490 cases were luminal A type, 1001 cases were luminal B type, 260 cases were HER-2 type, and 827 cases were TNBC type, showing a high heterogeneity.

**Table 1:** Main characteristics of the studies included in this meta-analysis

Study	Country	Number of patient	IHC evaluation	Cutoff value for PD-L1 positive	Positive cell (Tumor vs. immune)	HR for DFS	LL for DFS	UL for DFS	HR for OS	LL for OS	UL for OS
Qin T (2015)	China	870	Percentage	≥5%	tumor	1.503	1.091	2.071	2.262	1.598	3.203
Baptista M (2016)	Brazil	192	Allred score	≥4	tumor	0.84	0.39	1.83	0.3	0.09	0.94
Ali HR (2015)	England	5763	N/A	N/A	tumor and immune	N/A	N/A	N/A	N/A	N/A	N/A
Muenst S (2014)	Switzerland	650	H-score	≥100	tumor	N/A	N/A	N/A	4.43	3.424	5.731
Park IH (2016)	Korea	333	H-score	N/A	immune	1.21	0.56	2.62	2.08	0.86	5.04
Ghebeh H (2007)	Saudi Arabia	69	N/A	N/A	tumor	N/A	N/A	N/A	N/A	N/A	N/A

**Table 2:** Clinicopathologic parameters of the studies included in this meta-analysis

Study	Race	Age	Tumor size (2 cm ≤)	Tumor size (>2 cm)	HG1	HG2	HG3	LN (−)	LN (+)	ER (+)	ER (−)	PR (+)	PR (−)	HER-2 (+)	HER-2 (−)	KI-67 ≤ 14	KI-67 > 14
Qin T (2015)	Eastern Asian	median 47 (21-84)	282	57	37	338	495	427	443	630	240	617	253	18	852	123	440
Baptista M (2016)	N/A	N/A	57	132	33^a^		136	62	127	94	83	89	88	25	150	N/A	N/A
Ali HR (2015)	N/A	N/A	2739	2741	896	2270	2101	2823	2527	3086	1298	N/A	N/A	672	3805	N/A	N/A
Muenst S (2014)	N/A	median 64 (27-101)	181	469	143	259	248	355	294	457	191	N/A	N/A	129	519	118	525
Park IH (2016)	N/A	median 47 (28-78)	191	125	N/A	N/A	N/A	199	117	HR^b^ (+) 176	HR^b^ (−) 140	HR^b^ (+) 176	HR^b^ (−) 140	102	209	155	157

*HG* histologic grade, *LN* lymph node metastasis, *N/A* not applicable, *HR* hormonal receptor

^a^The histologic grade was classified as 1/2 and 3 in the study

^b^Hormonal receptor (+) was defined as ER (+) or PR (+) and hormonal receptor (−) was defined as ER (−) and PR (−) in the study

Most of the studies used a cross-sectional design to investigate PD-L1 expression in breast cancer, and univariate analyses to evaluate DFS and OS. Every study evaluated PD-L1 expression using immunohistochemistry, and most studies used a polyclonal rabbit anti-PD-L1 antibody (Abcam, Cambridge, MA). Four studies evaluated PD-L1 expression in tumor cells, 1 study evaluated immune cells (lymphocytes), and one study evaluated both tumor and immune cells. The positive cut-off values for the immunohistochemistry varied between the studies, with some studies evaluating the proportion of cells with positive staining, and other studies using the H-score and Allred score to evaluate both staining intensity and staining percentage.

### Associations of PD-L1 expression with clinicopathological parameters

The included studies evaluated various clinicopathological parameters, such as tumor size (≤2 cm vs. >2 cm), histological grade (1–2 vs. 3), lymph node metastasis, ER status, PR status, HER-2 status, Ki-67 labeling index, and molecular subtype (non-TNBC vs. TNBC). The studies all evaluated different cell populations for positive PD-L1 expression. Therefore, we analyzed PD-L1 positivity in all cells (tumor and immune cells) and in only tumor cells.

### PD-L1 expression in tumor and immune cells

**Fig. 2:**
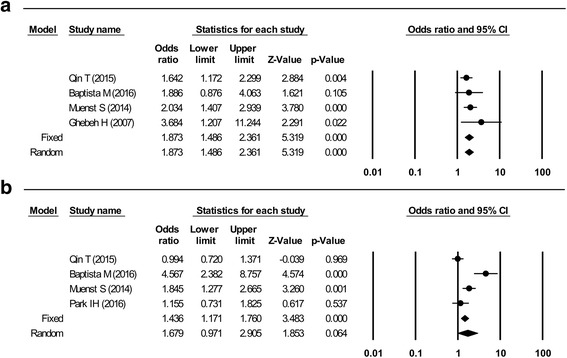
Forest plots of studies that assessed the association between PD-L1 and clinicopathological factors in all cells. **a** Histological grade. **b** Lymph node metastasis

Higher PD-L1 expression in all cells was associated with higher histological grade and lymph node metastasis. The pooled RR for higher histological grade was 1.87 (95% CI: 1.49–2.36, Z = 5.32, *p* < 0.001; Fig. 2a), and the fixed-effect model was used because of the low heterogeneity (I^2^ = 0%, *p* = 0.53). The pooled RR for lymph node metastasis was 1.68 (95% CI: 0.97–2.91, Z = 1.85, *p* = 0.06; Fig. 2b). Tumor size, ER status, PR status, HER-2 status, Ki-67 labeling index, and molecular subtype (non-TNBC vs. TNBC) were not significantly associated with PD-L1 expression in all cells.

### PD-L1 expression in only tumor cells

**Fig. 3:**
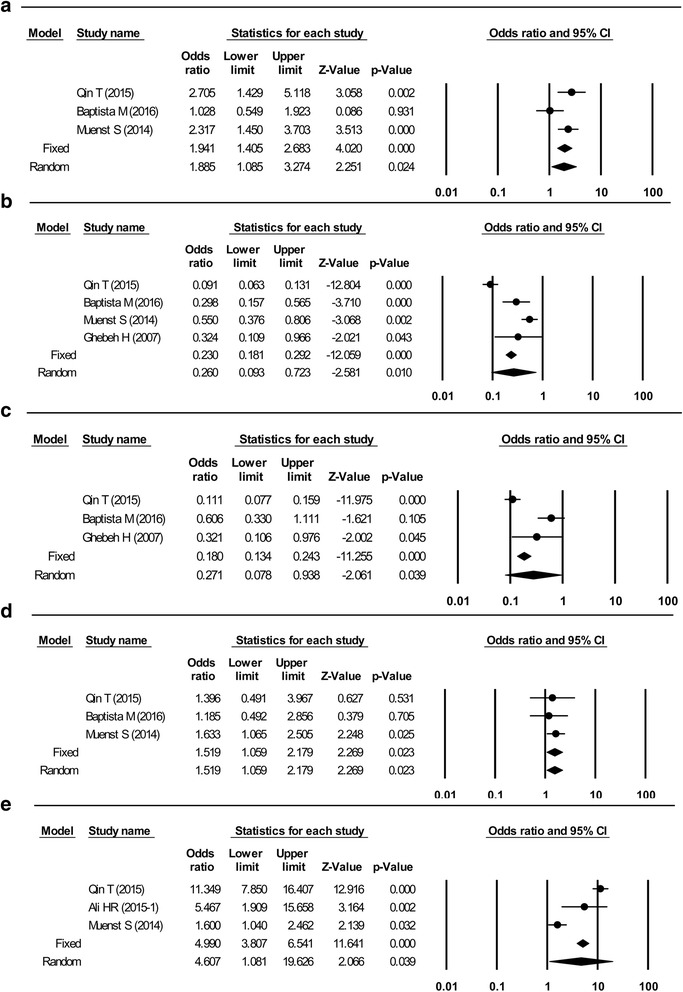
Forest plots of studies that assessed the association between PD-L1 and clinicopathological factors in tumor cells. **a** Tumor size. **b** Estrogen receptor status. **c** Progesterone receptor status. **d** Human epidermal growth factor receptor 2 status. **e** Molecular subtype

Higher PD-L1 expression in only tumor cells was associated with larger tumor size (pooled RR: 1.89, 95% CI: 1.09–3.27; Fig. 3a), ER negativity (pooled RR: 0.26, 95% CI: 0.09–0.72; Fig. 3b), PR negativity (pooled RR: 0.27, 95% CI: 0.08–0.94; Fig. 3c), HER-2 positivity (pooled RR: 1.52, 95% CI: 1.06–2.18; Fig. 3d), and TNBC (pooled RR: 4.61, 95% CI: 1.08–19.63; Fig. 3e). Most variables were assessed using a random-effect model, although a fixed-effect model was used for HER-2 status because of its low heterogeneity (I^2^ = 0%, *p* = 0.80). Histological grade, lymph node metastasis, and Ki-67 labeling index were not significantly associated with PD-L1 expression in only tumor cells.

### Effect of PD-L1 expression on survival (DFS and OS)

**Fig. 4:**
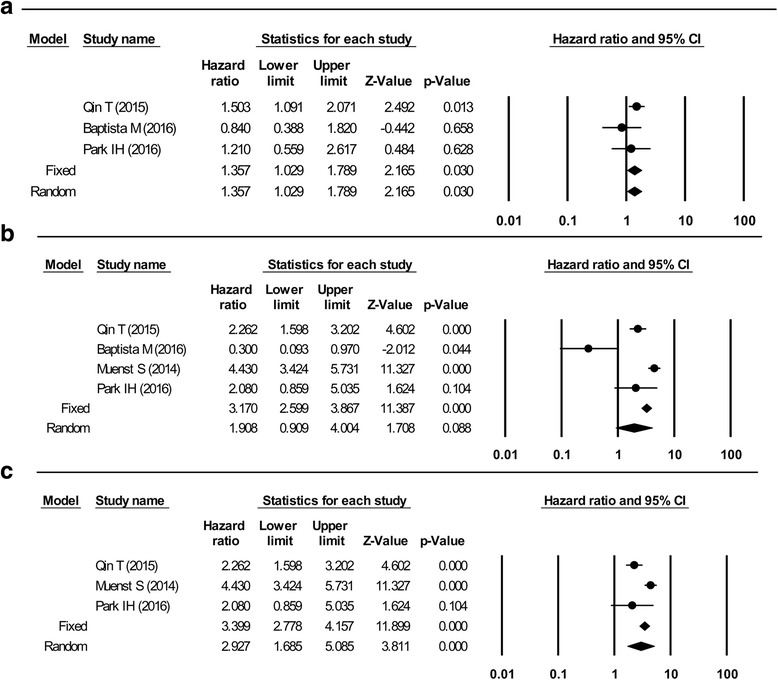
Forest plots of studies that assessed the association between PD-L1 and survival outcome in all breast carcinoma cells. **a** Disease-free survival. **b** Overall survival. **c** Overall survival without one study (Baptista et al. 2016, reference [11])

PD-L1 positivity in all cells was associated with poorer DFS, compared to PD-L1 negativity, although there was no significant difference in OS. The combined HR for DFS was 1.36 (95% CI: 1.03–1.79, *p* = 0.03; Fig. 4a), and low heterogeneity was detected in the included studies (*P* = 0.38, I^2^ = 0%). The combined HR for OS was 1.908 (95% CI: 0.91–4.00, *p* = 0.09; Fig. 4b), although significant heterogeneity was detected in the included studies (*p* < 0.001, I2 = 89%). When we re-performed the analysis after excluding the study by Baptista et al. [11], the combined HR for OS was 2.93 (95% CI: 1.69–5.09, *p* < 0.001) and significant heterogeneity was detected in the included studies (*p* = 0.005, I^2^ = 81%), although PD-L1 positivity now exhibited a significant association with poorer OS (Fig. 4c).

### Publication bias

**Fig. 5:**
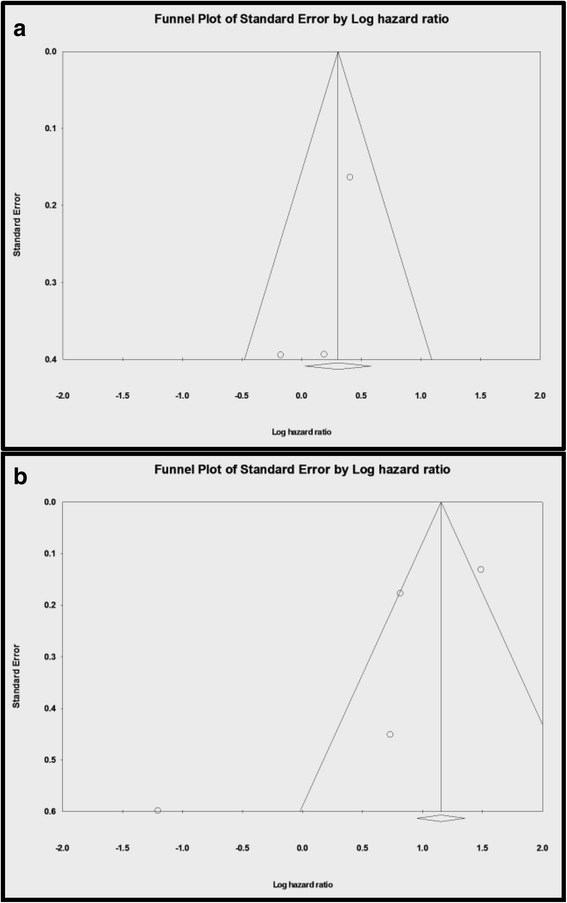
Egger’s test and funnel plot results for all included studies. **a** Overall survival based on all cells (*p* = 0.17). **b** Disease free survival based on all cells (*p* = 0.15)

The results from Egger’s test (*p* > 0.05) and the appearance of the funnel plot revealed that publication bias existed (Fig. 5).

## Discussion

Previous research has highlighted the importance of the tumor microenvironment, which includes non-tumor cells with non-transformed elements (in close proximity to tumor cells), immune cells (e.g., macrophages and lymphocytes), blood vessel cells, fibroblasts, myofibroblasts, mesenchymal stem cells, adipocytes, and the extracellular matrix. This information has led to the development of immunotherapy as an option for cancer treatment. In this context, PD-1 and PD-L1 play roles in a typical immune pathway, and PD-L1 is expressed in 20–70% of patients with lung cancer [4, 21–24], urinary bladder cancer [25], malignant melanoma [26], and ovarian cancer [27].

Several studies have evaluated PD-L1 expression in patients with breast cancer, although their conflicting results necessitated a meta-analysis. Therefore, the present meta-analysis aimed to evaluate the clinicopathological and prognostic significance of PD-L1 expression in breast cancer. Our results revealed that higher histological grade and lymph node metastasis were associated with higher PD-L1 expression in tumor and immune cells, and that PD-L1 expression in only tumor cells was associated with larger tumor size, higher histological grade, ER negativity, PR negativity, HER-2 negativity, and TNBC. Previous studies have referred to the relationship between higher histological grade, lymph node metastasis, larger tumor size, and PD-L1 positivity as the ‘immune escape’ phenomenon. In this context, cancer cells often express tumor antigens that are identified by the host immune system, which results in clearance. However, an insufficient immune response reduces the anti-tumor reaction in most cases (the immune escape) [1, 16, 28, 29]. In breast cancer, Fas-ligand-positive breast cancer cells induce the apoptosis of Fas-positive activated lymphocytes, which also results in immune escape [30]. Furthermore, activation of the PD-1/PD-L1 pathway lyses activated T-lymphocytes, which protects cancer cells from the host’s immune system [1, 31–33]. These relationships could be partially responsible for tumor development and progression, and are consistent with the findings of the present study, which revealed associations of poor prognosis with higher histological grade, lymph node metastasis, and larger tumor size. Furthermore, previous studies have suggested that there is a relationship between PD-L1 and TNBC, as TNBC exhibits increased peri-tumoral infiltration of CD8+ T-cells. This finding indicates that an abnormal immune pathway is involved in TNBC tumorigenesis, which might be related to higher PD-L1 expression in antigen-presenting cells [12, 34]. In addition, the present study revealed that PD-L1 positivity was associated with established predictors of a poor prognosis: ER negativity, PR negativity, and HER-2 negativity. Therefore, although the underlying mechanism remains elusive, the relationship between PD-L1 positivity and tumor aggressiveness may be related to the immune escape phenomenon. Nevertheless, further studies are needed to evaluate this possibility.

In the present study, PD-L1 expression in tumor or immune cells was associated with poorer DFS. Similarly, Sabatier et al. evaluated the expression of PD-L1 mRNA in 45 breast cancer cell lines and 5454 breast cancer cases [1], and found that higher PD-L1 mRNA expression was associated with larger tumor size, higher histological grade, ER and PR negativity, HER-2 positivity, high proliferation, and the basal and HER-2 subtypes (known markers of a poor prognosis). These findings suggest that PD-L1-positive cells are more invasive and have an aggressive phenotype, compared to other cells. In contrast, Baptista et al. found that PD-L1 positivity was associated with good OS [11], although their study included a larger proportion of ER-negative cases, compared to previous studies. Furthermore, previous studies of ER-negative breast cancer with PD-L1 positivity revealed a better survival rate [1, 12], which may indicate that the conflicting findings of Baptista et al. may be related to their case selection. Moreover, when we re-performed our analysis after excluding the results of Baptista et al., the combined HR for OS was 2.93 (95% CI: 1.69–5.09, *p* < 0.001) with significant heterogeneity in the included studies (*p* = 0.005, I^2^ = 81%). Thus, it remains possible that PD-L1 positivity is associated with poorer OS (Fig. 4c).

In PD-L1-positive cancer, targeting PD-L1 may help improve the antitumor immune response, and several recent preclinical and clinical trials have evaluated PD-L1-targeted therapy [21–23, 25, 35–37]. For example, two anti-PD-L1 antibodies have been developed: BMS-936559 [38] and MPDL3280A [22, 25]. BMS-936559 provided good efficacy in a study of various malignancies [38], which included tumor regression and the prevention of disease progression in non-small cell lung cancer, melanoma, and renal cell carcinoma. Another study evaluated patients with various advanced incurable cancers, and found that MPDL3280A provided confirmed responses (complete and partial response) in 18% of the patients [22]. Therefore, it may be important to evaluate PD-L1 expression in tumor cells, and the simplest and most convenient technique is immunohistochemistry using formalin-fixed paraffin-embedded specimens and a monoclonal anti-PD-L1 antibody. The commercially available monoclonal PD-L1 antibody clones are 28-8 [39], 22C3 [40], SP142 [22, 25], and E1L3N [41, 42]. In the present study, PD-L1 expression in all cells was associated with poorer DFS in breast cancer cases, which further highlights the possible therapeutic value of anti-PD-L1 therapy for breast cancer.

The present study has several strengths and limitations. The first strength is that, to the best of our knowledge, this is the first meta-analysis of PD-L1 expression and prognosis among patients with breast cancer. Second, we only included six studies, although these studies included a large patient population (7877 patients). Nevertheless, our findings should be interpreted with caution, based on their inherent limitations. First, there was strong publication bias among the included studies. This may have been caused by the heterogeneity of clinicopathologic characteristics, such as race, age, molecular genetic entities and tumor size, which resulted in a smaller effect in the meta-analysis. Second, as the clone and the manufacturer of the PD-L1 antibody that was used among the studies were different, this might have affected in different staining patterns and sensitivity. In particular, most studies included in this meta-analysis used rabbit anti-PD-L1 polyclonal antibodies (Abcam, Cambridge, MA). Compared to monoclonal antibodies, polyclonal antibodies have limitations that they could often show unspecific binding, high background staining and lack of reproducibility. Therefore, the difference in antibodies that were used might have influenced in the result of this study. Third, the cell components that were evaluated for PD-L1 staining and the thresholds that were used in the interpretation of PD-L1 positivity were different. Therefore, future studies are needed to prospectively evaluate a large group of patients using a standardized assessment of PD-L1 staining, which may help validate our findings.

## Conclusions

Our meta-analysis revealed that PD-L1 positivity in tumor or immune cells from patients with breast cancer was significantly associated with higher histological grade, lymph node metastasis, and poorer DFS. Therefore, positive PD-L1 expression may be useful for predicting prognosis among patients with breast cancer.

## Additional file

Additional file 1: The primary characteristics of the included studies. The primary characteristics of the included studies. (XLSX 22 kb)

## Abbreviations

CI: Confidence interval
DFS: Disease-free survival
ER: Estrogen receptor
HER-2: Human epidermal growth factor type 2
HR: Hazard ratio
OS: Overall survival
PD-1: Programmed cell death protein 1
PD-L1: Programmed cell death-ligand 1
PR: Progesterone receptor
RR: Relative risk
TNBC: Triple-negative breast cancer
